# Seroprevalence of *Encephalitozoon cuniculi* and *Toxoplasma gondii* antibodies and risk-factor assessment for *Encephalitozoon cuniculi* seroprevalence in Finnish pet rabbits (*Oryctolagus cuniculus*)

**DOI:** 10.1186/s13028-022-00622-5

**Published:** 2022-02-02

**Authors:** Johanna Mäkitaipale, Emmi Järvenpää, Anne Bruce, Satu Sankari, Anna-Maija Virtala, Anu Näreaho

**Affiliations:** 1 Department of Equine and Small Animal Medicine, Faculty of Veterinary Medicine, University of Helsinki, P.O. Box 57, 00014 Helsinki, Finland; 2 Department of Veterinary Biosciences, Faculty of Veterinary Medicine, University of Helsinki, P.O. Box 66, 00014 Helsinki, Finland

**Keywords:** ELISA, Encephalitozoonosis, IgG, Multivariable logistic regression, Parasitology, Toxoplasmosis

## Abstract

**Background:**

Neurological signs, such as head tilt, torticollis, paralysis, and seizures, are common in rabbits. Differential diagnoses include two zoonotic infections caused by the microsporidial fungi *Encephalitozoon cuniculi* and the apicomplexan protozoa *Toxoplasma gondii*. Both infections are mainly latent in rabbits but may cause severe or even fatal disease. Although several international studies have reported the seroprevalence of these pathogens in different commercial rabbit populations, similar prevalence studies and risk-factor analyses among family-owned pet rabbits are uncommon and lacking in Scandinavia. We sought to estimate the seroprevalence and possible risk factors for *E. cuniculi* and *T. gondii* among Finnish pet rabbits. We used ELISA to measure *E. cuniculi* IgG seroprevalence of 247 rabbits and modified direct agglutination test for *T. gondii* seroprevalence of 270 rabbits. Samples were collected as part of the Finnish Pet Rabbit Health Research project. Internet-based questionnaires (n = 231) completed by the rabbit owners were used for risk-factor analysis.

**Results:**

The apparent seroprevalence of *E. cuniculi* was 29.2% and true seroprevalence of *T. gondii* 3.9%. Risk factors were analysed only for *E. cuniculi* due to the low *T. gondii s*eroprevalence. The final multivariable logistic regression model revealed that rabbits spending the whole summer outdoors had a higher risk of being *E. cuniculi* seropositive than rabbits with limited outdoor access. Additionally, rabbits living in households with only one or two rabbits had higher risk of being *E. cuniculi* seropositive than those in multi-rabbit households.

**Conclusions:**

Nearly one third of Finnish pet rabbits participating in this study had *E. cuniculi* IgG antibodies, indicating previous exposure to this pathogen. The prevalence is similar to that reported previously in clinically healthy rabbit populations in UK and Korea. While the seroprevalence of *T. gondii* was low (3.9%), antibodies were detected. Therefore, these zoonotic parasitic infections should be considered as differential diagnoses when treating rabbits.

## Background

Neurological disorders are common in pet rabbits [1, 2]. Differential diagnoses include zoonotic infections caused by *Encephalitozoon cuniculi* and *Toxoplasma gondii* [2].

*Encephalitozoon cuniculi* is an obligate intracellular microsporidial fungi from the phylum Microspora and is widespread among wild and domestic rabbit populations around the world. Infection is mostly asymptomatic but can cause a disease called encephalitozoonoosis. *E. cuniculi* is most common in rabbits but may infect many other mammals including rodents, horses and carnivores, and even immunocompromised humans [3–6]. Granulomatous meningoencephalitis, granulomatous nephritis, and cataract followed by phacoclastic uveitis are the main severe and even fatal manifestations of this disease in rabbits [7–10]. Clinical signs are due to the foci of nonsuppurative granulomatous inflammation in lesions with primary location in the central nervous system, kidneys, and eyes [7, 8]. High IgG seroprevalence has been reported in asymptomatic rabbits, suggesting latent or chronic disease [7]. Diagnosis of acute infection in symptomatic rabbits is mainly based on analysing serum *E. cuniculi* IgG and IgM antibody levels [11]. IgM antibody levels increase in acute infection, whereas IgG levels indicate chronic or resolved infection [11].

Infected rabbits excrete spores of *E. cuniculi* in their urine and infection of new hosts can occur via direct contact with the urine or from spores present in the environment [6]. Spores survive in optimal environmental conditions for even 3 months. Survival time of 1 day at − 20 °C, 98 days at 4 °C, 6 days at 22 °C were reported for spores in medium 199 (without serum) [12]. After ingestion, spores are disseminated via blood flow to internal organs. Transplacental transmission of *E. cuniculi* is a possible source of infection for rabbit fetus [13, 14]. If the transplacental transmission of *E. cuniculi* occurs during the first trimester period of gestation when embryological development of lens capsule is active, ocular encephalitozoonosis and cataract may follow [14].

*Toxoplasma gondii* is prevalent in mammals and birds in most areas of the world [15]. It is an obligate intracellular protozoal coccidian parasite. Felids are definitive hosts and all warm-blooded animals, including rabbits and humans, can acts as intermediate hosts [16]. In Finland, high *T. gondii* seroprevalence (48.4%) has been reported in cats [17]. In humans, infection is mostly asymptomatic but can cause severe ocular toxoplasmosis and also be fatal in immunocompromised individuals and in neonates acquiring congenital infection transplacentally [18]. Consumption and handling of raw and undercooked rabbit meat are recognized as common risks factors for *T. gondii* infection in humans and owning a rabbit has, in previous study, been recognised as a risk factor for infection [19, 20]. Rabbit owners were suspected to become infected when handling hay contaminated with *T. gondii* oocysts from feline faeces, or by inhaling or ingesting oocysts while spending time outside in rabbit keeping facilities, where cats may roam freely [19]. Other possible, but rare modes of transmission include blood transfusions and organ transplants [18]. A few clinical case reports of fatal toxoplasmosis in domestic rabbits exist [21, 22], although infection is most often subclinical and latent [2]. Fever, lethargy, diarrhoea, dehydration, and peracute death without clinical signs have been reported in rabbits with fatal toxoplasmosis [21, 22]. Adult rabbits may be exposed to this pathogen through ingestion of contaminated food or water. Congenital infection is possible also in rabbits [23]. Good hygiene and prevention of the infection in breeding animals are therefore essential. Toxoplasmosis is usually fatal in European brown hares and mountain hares [24].

Although several international studies have reported the seroprevalence of *E. cuniculi* and *T. gondii* in different commercial rabbit populations, similar prevalence studies and risk-factor analyses among family-owned pet rabbits remain uncommon and are lacking in Scandinavia. The aim of this study was to determine the IgG seroprevalence of *E. cuniculi* and *T. gondii* in family-owned healthy pet rabbits. We also studied risk factors for the seroprevalence. We hypothesized regarding our clinical experience that the seroprevalence of *E. cuniculi* IgG antibodies would be approximately 20–25% as previously reported in healthy pet rabbits (in UK and Korea (23.1 and 25.8%, respectively [25, 26]) and that *T. gondii* seroprevalence is low but higher in families with pet cats. Knowledge on the seroprevalence of these infections is important for practicing veterinarians who treat rabbits, as rabbits are the third most common pets in many countries and owners are increasingly willing to treat them.

## Methods

Blood samples used in this study were collected between May 2012 and March 2014 as part of the Finnish Pet Rabbit Health Research Project at the Veterinary Teaching Hospital of the University of Helsinki. The study was approved by the Animal Experiment Board. Advertising of the study was done via internet and among the Finnish pet rabbit society. Owners registered their rabbits to the study by email on a voluntary basis, thus making sampling non-randomised. Only clinically healthy pet rabbits were included in the study.

Blood samples (1 mL per sample) were collected from the *vena cephalica* and from the *v. saphena* to plain serum tubes. After clotting, samples were centrifuged at 1485×*g* for 10 min. Sera were collected and stored at − 80 °C until used. Owners completed an electronic questionnaire (Eduix Oy, version 3, https://e-lomake.fi) (n = 231) regarding rabbits’ sex, breed, date of birth, place of origin (breeder, pet shop, rescue centre, or somewhere else), purpose of ownership (pet, hobby, breeding, or other), number of rabbits in the household, any interaction with other rabbits (yes/no), access to outdoors during summer months (May–August; daily/weekly/monthly/few times during summer/whole summer outdoors/never), cats in the house (yes/no), type of housing (inside a house as house-rabbit, outside in the garden, outside in a stable/barn/shed, somewhere else), if the rabbit ever had neurological signs (yes/no), and feeding frequency of selected diets (daily/weekly/monthly/seldom/never; wild plants, salads, vegetables, root vegetables, twigs). This questionnaire has been previously used in other studies [27–30].

We used OpenEpi [31] for sample size estimation. While the exact number of pet rabbits in Finland is unknown, we have used an estimation of 120,000 rabbits in our previous study [27]. In calculations, we estimated the seroprevalence of *E. cuniculi* to be between 20 and 25% using 95% confidence level. This yielded a sample size of 246 to 288 rabbits. To estimate the sample size needed for *T. gondii* seroprevalence, we estimated the seroprevalence at 95% confidence level, we estimated the seroprevalence to be 5–10%. This yielded a sample size of 73–139 rabbits.

We used a commercial enzyme-linked immunosorbent assay (*Encephalitozoon cuniculi* Antibody ELISA Test Kit, Medicago AB, Uppsala, Sweden) to analyse *E. cuniculi* seroprevalence from 247 rabbit serum samples. This assay was selected as it was previously used in several rabbit studies [8, 26, 32, 33]. The sensitivity and specificity of this kit were unknown. ELISA was performed following the manufacturer’s instructions. Serum samples were diluted 1:40 with phosphate buffered saline (PBS). Conjugate was diluted 1:100 with PBS and positive and negative controls were diluted 1:100 with PBS. After dilution, samples, conjugate, negative control, and positive control were dispensed into the antigen-coated 96-well microtiter plates. Positive and negative controls were run in each plate. Absorbances were read at 450 nm using a microplate reader (Wallac 1420 Victor2 1420, Perkin Elmer, Waltham, Massachusetts, US). ELISA results were calculated using the manufacturer’s protocol [(Sample A_450_ *E. cuniculi* coated − Sample A_450_ control antigen coated)/(Negative control A_450_ *E. cuniculi* coated − Negative control A_450_ antigen coated)]. Values > 2 were classified as positive for *E. cuniculi* antibodies.

A modified direct agglutination test (Toxo-Screen DA, bioMérieux SA, Marcy-l’Etoile, France) was used for screening of *T. gondii* IgG antibodies from 270 serum samples following the manufacturer’s instructions. This test was previously used in several rabbit studies [34, 35] and for other species in our laboratory [17, 24, 36]. The previously reported sensitivity and specificity of this kit were 100% and 94.8%, respectively [37]. Serum samples were diluted 1:40 and 1:4000 with PBS containing 2-mercaptoethanol. Each 96-well microtiter plate included positive and negative controls and antigen control. The plates were read after 18 h of incubation at room temperature in a stable place. The test was considered negative (IgG antibodies < 4 IU/mL) if both 1:40 and 1:4000 dilutions were negative and positive if 1:40 dilution was positive and 1:4000 dilution was negative, borderline, or positive.

### Statistical methods

Data were imported from the electronic questionnaire into SPSS statistical program (IBM SPSS version 25, Chicago, USA). Several independent variables were recategorized for statistical analyses. Rabbits were divided into two groups by age using 3 years as a cut-off point; in an earlier study we observed that rabbits > 3 years had more health problems than younger rabbits [38]. Breed was categorized into mixed-breed rabbits, Dwarf Lops, and others as these two breed groups were the most common.

Many variables initially had so many categories that only a few observations were made in some of the categories. Therefore, original categories were combined. We defined a household as a multi-rabbit household if it featured three or more rabbits and compared those to households with one or two rabbits. The origin of the rabbits was categorized to those from breeders and of other origin. The use of the rabbits was grouped for pet use and for other use. The living conditions were categorized into house rabbits and non-house rabbits. Access to outdoors was categorized to those spending the entire summer outdoors and to others (access to outdoors daily/weekly/monthly/few times during summer). The frequency of receiving wild plants, salads, vegetables, root vegetables, herbs, fruits, or twigs was divided into three categories (daily/occasionally/never) and for regression analyses into two categories (daily/occasionally or never) and salads also into ‘given ever or not’.

The prevalence of *T. gondii* and *E. cuniculi* seropositivity was determined; for *E. cuniculi* in all variable groups (Table 1). Seropositivity for *T. gondii* was not determined in variable groups due to low prevalence. The true prevalence for *T. gondii* was estimated from the obtained apparent prevalences with the sensitivity and specificity values of the test [37] with Epitools [39] using Blaker’s confidence interval (CI) [40]. Sensitivity and specificity of *E. cuniculi* test was unknown and true prevalence could not be therefore calculated. EpiTools [39] was used to calculate the 95% CI for the seroprevalence *of E. cuniculi* in the study population applying the Wilson’s method [41]. The association between age in years and if the rabbit was from a multirabbit household or from a household with one or two rabbits was studied using independent samples Mann–Whitney *U* test.

**Table 1:** Apparent seroprevalence of *Encephalitozoon cuniculi* in Finnish pet rabbits in various suspected risk-factor subgroups

Factor	Subgroup	Total (n)^a^	Seropositive (n)	Prevalence (%)	OR^b^ (95% CI^c)^	P value^d^
Age with a 3-year cut-off	< 3 year	129	29	22.5	Ref^e^	
	≥ 3 year	89	36	40.4	2.3 (1.3–4.2)	0.005
Sex	Female	107	34	31.8	Ref	
	Male	111	30	27.0	0.8 (0.4–1.4)	0.442
Neutered	Yes	89	26	29.2	0.9 (0.6–1.9)	0.918
	No	126	36	28.6	Ref	
Breed	Mixed breed	62	19	30.6	1.3 (0.7–2.6)	0.464
	Dwarf LOP	55	19	34.5	1.6 (0.8–3.1)	0.224
	Other	110	28	25.5	Ref	
Bought from a breeder	Yes	127	34	26.8	0.9 (0.5–1.6)	0.636
	No	84	25	29.8	Ref	
Usage	Pet	146	43	29.5	1.1 (0.6–2.1)	0.767
	Breeding or hobby	62	17	27.4	Ref	
Living as a house rabbit	Yes	147	41	27.9	0.9 (0.5–1.7)	0.700
	No	69	21	30.4	Ref	
Housing	Multi-rabbit household	104	22	21.2	0.5 (0.3–0.9)	0.032
	One- or two-rabbit household	82	29	35.4	Ref	
Meets other rabbits	Yes	128	29	22.7	0.5 (0.3–0.9)	0.016
	No	87	33	37.9	Ref	
Cats in the family	Yes	58	18	31.0	1.1 (0.6–2.1)	0.761
	No	166	48	28.9	Ref	
Whole summer outdoors	Yes	35	17	48.6	3.1 (1.4–6.8)	0.004
	No	133	31	23.3	Ref	
Given wild plants daily	Yes	55	16	29.1	1.0 (0.5–2.0)	0.911
	No	159	45	28.3	Ref	
Ever given salads	Yes	191	51	26.7	0.4 (0.2–0.9)	0.027
	No	22	11	50.0	Ref	
Given root vegetables daily	Yes	110	26	23.6	0.6 (0.3–1.0)	0.050
	No	109	39	35.8	Ref	
Given twigs daily	Yes	63	23	36.5	1.6 (0.9–3.0)	0.149
	No	154	41	26.6	Ref	
Given vegetables daily	Yes	45	15	33.3	1.3 (0.6–2.6)	0.505
	No	170	48	28.2	Ref	
Given herbs daily	Yes	20	6	30.0	1.0 (0.4–2.8)	0.981
	No	195	58	29.7	Ref	
Given fruits daily	Yes	30	6	20.0	0.6 (0.2–1.4)	0.212
	No	185	58	31.4	Ref	
Neurological signs^f^	Yes	11	6	54.5	3.2 (0.9–11.1)	0.063
	No	177	48	27.1	Ref	
*Toxoplasma* seropositivity	Yes	7	3	42.9	2.0 (0.4–8.9)	0.392
	No	230	64	27.8	Ref	

^a^n = number of rabbits

^b^OR = Odds ratio obtained with unconditional logistic regression analysis associations between the factor and being *E. cuniculi* seropositive

^c^CI = confidence interval

^d^Wald’s P value for OR

^e^Ref = reference group

^f^At least once in the rabbit’s lifetime

Preliminary associations between the outcome (*E. cuniculi* seropositive or seronegative) with all variables were calculated using Fisher’s exact test and unconditional logistic regression analyses (with only one variable in the model at a time). Those with P < 0.2 were entered into multivariable logistic regression. The model selection was performed by backward selection. Significance was considered at α = 0.05 level. Goodness of fit of the model was evaluated with Omnibus and Hosmer-Lemenshow tests. The model prediction was evaluated with percentage accuracy in classification, sensitivity and specificity, and discrimination with area under the curve (AUC) of the receiver operating characteristic (ROC) curve. All two-level interactions between the variables in the final model were tested. Multicollinearity between variables was estimated by Phi coefficient; the limit value for strong correlation was set at 0.5 [42]. We tested for confounding by including the most potential confounding variables (‘sex’, ‘age’, and ‘breed’) into the model and observed their effects on the odds ratios (OR) of the remaining variables. The confounder was included in the model even if it was not statistically significant if it changed the OR approximately 20% or more.

## Results

Mean age of the rabbits was 3.1 years (range 0.15–11.8, median 2.5 years). Mixed-breed rabbits were the most common (n = 65, 23.2%), followed by Dwarf Lops (n = 64, 22.9%) (Table 1). Altogether 24 different rabbit breeds were represented (data on breed missing in 25 cases, 8.9%). Rabbits from multi-rabbit households were significantly younger than those from a household with one or two rabbits (P = 0.001).

Apparent *E. cuniculi* seroprevalence was 29.2% (72/247; 95% CI 23.8–35.1%). Seroprevalence values in different suspected risk-factor subgroups are presented in Table 1. The final multivariable logistic regression model indicated that rabbits spending the whole summer outdoors had almost eight-fold higher odds of being *E. cuniculi* seropositive than rabbits with limited daily/weekly/monthly/few times during summer access to outdoors. Rabbits in households with one or two rabbits also had four-fold greater odds of being *E. cuniculi* seropositive than those in multi-rabbit households (Table 2). This model was significant (Omnibus test χ^2^(4) = 20.3, P < 0.0005); the Hosmer-Lemenshow test was not statistically significant (P = 0.123), indicating that the model is not poor fit. The model explained 18.7% (Nagelkerke R^2^) of the variation in *E. cuniculi* seropositivity in this study population consisting of asymptomatic pet rabbits, classified 74.3% of rabbits correctly into being seropositive or seronegative, and had 98% specificity but only 16.7% sensitivity. The model had acceptable discrimination based on AUC (0.7; 95% CI 0.6–0.8) [43]. Breed was a confounder and was kept in the model although it was non-significant, as it changed the OR of the remaining variables over 20%. Interaction was not detected. There was no association in this study population between being outside the whole summer and the household of the rabbit (1–2 rabbits vs. multi-rabbit household).

**Table 2:** Final multivariable logistic regression model for *Encephalitozoon cuniculi* seropositivity in Finnish pet rabbits

Risk factor	b^a^	Wald’s P	OR^b^ (95% CI^c^)
One to two rabbits in household vs at least three	1.4	0.003	4.1 (1.6–10.2)
Whole summer outdoors vs not	2.1	< 0.0005	7.9 (2.8–22.4)
Breed	NA^d^	0.200	NA
Mixed	− 0.5	0.300	0.6 (0.2–1.6)
Dwarf lop	0.5	0.279	1.7 (0.6–4.5)
Other	NA	NA	Ref^e^
Constant	− 2.1	< 0.0005	NA

^a^Regression coefficient

^b^Odds ratio

^c^95% confidence interval for OR

^d^Not applicable

^e^Reference group

The true seroprevalence of *T. gondii* was 3.9% (8/270; 95% CI 3.5–4.1%). Three out of seven *T. gondii*-positive rabbits were also *E. cuniculi* positive (42.9%; 95% CI 15.8–75.0) (*E. cuniculi* test result was missing in one *T. gondii*-positive rabbit). Mean age of *T. gondii*-positive rabbits was 2.7 years (range 1.0–6.9). Five were female and two were male. One rabbit was from a rescue centre, two were re-housed from the previous owner, and the remainder were from a breeder. Three rabbits lived inside a house as house rabbits, two in an outbuilding, two outside in a garden, and one rabbit lived in a balcony. All except one rabbit had access to outdoors during summer months; three spent the whole summer outdoors, one had daily outdoor access, two had weekly outdoor access, and one a few times during the summer. There were cats in families of 3/8 T*. gondii*-positive rabbits. Only one rabbit of these was also *E. cuniculi* positive.

Three out of seventy (4.3%; 95% CI 1.2–11.9%) *E. cuniculi-*positive rabbits were *T. gondii* seropositive, while 4 out of 177 (2.3%; 95% CI 0.9–5.7%) *E. cuniculi*-negative rabbits were *T. gondii* seropositive. Only three rabbits (3/237, 1.3%; 95% CI 0.4–3.7%) were seropositive for both pathogens.

## Discussion

Nearly 30% of the pet rabbits participating in this study had *E. cuniculi* IgG antibodies. As the sampling was not randomised due to voluntary participation, the prevalence may not represent the prevalence in all Finnish rabbits. This result is consistent with the results of previous studies of asymptomatic pet rabbits in UK and Korea (23.1 and 25.8%, respectively [25, 26]) but was much lower compared to the seroprevalence observed in another study of healthy domestic rabbits in the UK (51.5%, [44]), asymptomatic pet rabbits in Italy (68.1% [32]; 52.9%, [45]), and asymptomatic pet rabbits in Brazil (85.0%, [46]). The highest seroprevalence values were reported in rabbits with neurological symptoms typical of *E. cuniculi* infections (70.65% [45], 86.1% [11]) and in commercially reared asymptomatic breeding does in large Italian farms with in total 300–3000 breeding does and 2000–14,000 growing rabbits [48]. Lower *E. cuniculi* seroprevalence compared to our results have been reported in rabbits from commercial breeding and farming facilities (20.5% in Czech and Slovak Republics [47], 19.4% in China [33]). Several factors may explain the differences between seroprevalences of various study populations. Baldotto et al. [46] listed four factors to explain the high seroprevalence observed in their study of Brazilian pet rabbits: (1) tropical and subtropical climate, (2) owners´ lack of knowledge of the disease and its transmission, (3) poor general husbandry practices, and (4) presence of same rabbit family with several generations in same household resulting in transplacental exposure. Climate conditions in northern countries with cold winter as in Finland, reduce the infectiveness of the spores, but also restrict the possibility to keep rabbits outdoors. Voluntary participation to this study might have affected to participation of more keen rabbit-owners compared to those with less knowledge regarding rabbit diseases. Although there was high heterogeneity in the living conditions of the rabbits in this study, 60% of the rabbits lived inside as house rabbits. House rabbits usually have separate litter boxes making cleaning easier as urine and feaces do not contaminate the entire living area. Also, the smell of these might activate the owner of house rabbits to clean the boxes more often compared to owners of non-house rabbits. Therefore the environmental hygiene might be good for the rabbits of our study and may additionally explain the low seroprevalence.

Due to the low seroprevalence of *T. gondii*, risk factors were analysed only for *E. cuniculi* infections. The final multivariable logistic regression model revealed that rabbits spending the whole summer outdoors had higher risk of being *E. cuniculi* seropositive than rabbits with limited outdoor access. It is possible that hygiene in the outdoor shelters and hutches are poorer than those inside a house and this may increase the risk for infection. Wild rabbits exist in Southern Finland and may spread *E. cuniculi* spores via urine to grass and other outdoor vegetation. Pet rabbits spending the whole summer outdoors could potentially get infection from them.

Surprisingly, rabbits in the households with one or two rabbits had higher risk for being *E. cuniculi* seropositive than rabbits in multi-rabbit households. Hygiene and overall care were expected to be better in the households with one or two rabbits than in multi-rabbit households and would therefore decrease the risk of being seropositive. However, rabbits in multi-rabbit households were significantly younger than rabbits in households with one or two rabbits, which may explain this finding. We analysed IgG antibodies indicating a previously acquired infection. Older rabbits had a longer period to become infected than younger rabbits. It is also possible that the rabbits were infected in utero or postnatally within the first 8 weeks of life while still living in breeding facilities and less frequently later in life if living in conditions with good hygiene. Unfortunately, we did not include a question regarding possible previous 28-day treatment with fenbendazole for *E. cuniculi* infection in our questionnaire. Routine *E. cuniculi* treatments with fenbendazole are not generally recommended for asymptomatic rabbits in Finland, but may be more commonly used in multi-rabbit households to prevent outbreaks of clinical encephalitozoonoosis in breeding and hobby rabbits. It is unknown how long rabbits remain *E. cuniculi* seropositive. Therefore, prospective long-term or even lifetime follow-up studies are necessary to further study this subject.

Feeding frequency of the selected diets was not associated with increased risk for *E. cuniculi* seropositivity. It is possible that rabbits acquire the infection from contaminated hay, water, or the environment instead of these selected diets.

Previously, Lonardi et al. [48] performed a risk-factor analysis using logistic regression and identified that hybrid X rabbits and rabbits older than 120 days had a higher risk of being *E. cuniculi* seropositive than rabbits of other breeds or younger rabbits. There was high variation in rabbit breeds in our study and most rabbits were of pet rabbit breeds. Therefore, we categorized the rabbits as mixed-breed rabbits, Dwarf Lops, and others. Although breed did not have a significant effect in our study, it was included in the model due to its confounding effect. While the seroprevalence was higher in rabbits > 3 years and older rabbits had a greater risk for *E. cuniculi* seropositivity in unconditional logistic regression analysis, age did not remain in the final multivariable logistic regression model.

The *T. gondii* seroprevalence in our study population was lower than expected, as only 8 out of 270 (3.9%) rabbits were positive. The seroprevalence in Finnish wild rabbits has not yet studied, but the 4.2% seroprevalence was reported in Finnish mountain hares [24]. Toxoplasmosis was the cause of death in 8.1% of European brown hares and 2.7% of mountain hares in post-mortem studies in Finland [24]. The observed prevalence in our study is low when compared to the reviewed seroprevalence levels of 10–15% in domestic and wild rabbits [49]. A seroprevalence of 8.9% was reported in healthy Polish pet rabbits [35]) and in 0.9% of Japanese pet rabbits [50]. The seroprevalence of *T. gondii* in Japanese cats was 5.4% [51], which is much lower than that of Finnish cats (48.4%, [17]); this could also explain the difference in seroprevalence between Japanese and Finnish rabbits. Only three out of eight *T. gondii*-positive rabbits in our study were living in a household with cats. However, seven of the *T. gondii*-positive rabbits had access to outdoors during summertime (data missing in one case), and all except one were fed with outdoor greens at least a few times weekly. Grass and other plants contaminated with cat faeces may have been the source of infection for these rabbits. Sporulated oocysts of *T. gondii* may survive in the environment for months or even years [14]. Invertebrates (such as flies) can mechanically spread oocysts [14], which may be a source of infection even for a rabbit living inside a house. Pet rabbits that were fed unwashed vegetables had a higher seroprevalence than those fed washed vegetables in a Polish study [35]. This study also revealed that rabbits having contact with cats were more commonly *T. gondii* seropositive than rabbits without cat exposure [35].

Neumayerová et al. [47] reported higher *T. gondii* seroprevalence in *E. cuniculi*-seropositive rabbits than *E. cuniculi*-seronegative rabbits. In our study, the low number of *T. gondii*-seropositive rabbits limited the possibilities to further study these coinfections.

Although we collected the minimum planned sample size required for *E. cuniculi* seroprevalence, a larger sample size would have been necessary due to the slightly higher observed seroprevalence. We also managed to exceed the sample size required for *T. gondii* seroprevalence and this was a strength of this study. Due to the low seroprevalence of *T. gondii*, we were unable to perform a risk-factor analysis for *T. gondii* seroprevalence. As the *E. cuniculi* seroprevalence was not adjusted with the sensitivity and specificity of the test used, the seroprevalence is presented as apparent seroprevalence. We used owner questionnaires to obtain the rabbits’ background information and some information was therefore missing. Use of owner questionnaires includes a risk of information bias. Although the questionnaire was not validated prior to use, it was used in few other studies previously. We did not have any information about the geographical region of the rabbits wherefore we could not adjust for that in the analyses. As climate conditions during winter in whole Finland are not beneficial for *E. cuniculi* spores’ stability and *T. gondii* oocysts’ sporulation, we did not find the information regarding the region as important.

## Conclusions

Nearly one-third of Finnish pet rabbits participating in this study were seropositive for *E. cuniculi* IgG antibodies, indicating previous exposure to this pathogen. Seroprevalence of *T. gondii* was low (3.9%). These zoonotic infections should be considered as differential diagnoses when treating rabbits in veterinary clinics.

## Data Availability

The datasets used and analysed in the current study are available from the corresponding author on reasonable request.
